# Antiretroviral Therapy Reduces HIV Transmission in Discordant Couples in Rural Yunnan, China

**DOI:** 10.1371/journal.pone.0077981

**Published:** 2013-11-13

**Authors:** Na He, Song Duan, Yingying Ding, Keming Rou, Jennifer M. McGoogan, Manhong Jia, Yuecheng Yang, Jibao Wang, Julio S. G. Montaner, Zunyou Wu, for the China National HIV Prevention Study Group

**Affiliations:** 1 Department of Epidemiology, School of Public Health, and the Key Laboratory of Public Health Safety of Ministry of Education, Fudan University, Shanghai, China; 2 Dehong Prefecture Center for Disease Control and Prevention, Mangshi, Yunnan Province, China; 3 National Center for AIDS/STD Control and Prevention (NCAIDS), Chinese Center for Disease Control and Prevention (China CDC), Beijing, China; 4 Yunnan Provincial Center for Disease Control and Prevention, Kunming, Yunnan Province, China; 5 British Colombia Centre for Excellence in HIV/AIDS, St Paul's Hospital, and Division of AIDS, Faculty of Medicine, University of British Columbia, Vancouver, Canada; University of Washington, United States of America

## Abstract

**Background:**

Although HIV treatment as prevention (TasP) via early antiretroviral therapy (ART) has proven to reduce transmissions among HIV-serodiscordant couples, its full implementation in developing countries remains a challenge. In this study, we determine whether China's current HIV treatment program prevents new HIV infections among discordant couples in rural China.

**Methods:**

A prospective, longitudinal cohort study was conducted from June 2009 to March 2011, in rural Yunnan. A total of 1,618 HIV-discordant couples were eligible, 1,101 were enrolled, and 813 were followed for an average of 1.4 person-years (PY). Routine ART was prescribed to HIV-positive spouses according to eligibility (CD4<350 cells/µl). Seroconversion was used to determine HIV incidence.

**Results:**

A total of 17 seroconversions were documented within 1,127 PY of follow-up, for an overall incidence of 1.5 per 100 PY. Epidemiological and genetic evidence confirmed that all 17 seroconverters were infected via marital secondary sexual transmission. Having an ART-experienced HIV-positive partner was associated with a lower rate of seroconvertion compared with having an ART-naïve HIV-positive partner (0.8 per 100 PY *vs.* 2.4 per 100 PY, HR = 0.34, 95%CI = 0.12–0.97, p = 0.0436). While we found that ART successfully suppressed plasma viral load to <400 copies/ml in the majority of cases (85.0% *vs.* 19.5%, p<0.0001 at baseline), we did document five seroconversions among ART-experienced subgroup.

**Conclusions:**

ART is associated with a 66% reduction in HIV incidence among discordant couples in our sample, demonstrating the effectiveness of China's HIV treatment program at preventing new infections, and providing support for earlier ART initiation and TasP implementation in this region.

## Introduction

At the end of 2011, approximately 780,000 people were living with HIV/AIDS and heterosexual transmission remained the primary mode of transmission in 47% of cases in China [1]. We also witnessed a rise in proportion of new infections in people that engage in stable, HIV-serodiscordant relationships. Hence, individuals in serodiscordant relationships represent a growing high-risk population [1]–[5].

Antiretroviral therapy (ART) is increasingly recognized as a very promising strategy for reducing HIV transmission among discordant couples [6], [7]. Effective viral suppression through ART has been associated with a significant reduction in infectivity and therefore diminished risk of HIV transmission in studies conducted in North America [8], [9], Africa [5], [10], [11], Europe [12], and South America[13]. The benefit of ART in preventing new HIV infections has been confirmed by the HIV Prevention Trials Network (HPTN) 052 study, a two-arm, multi-site (nine countries) randomized trial on the effectiveness of treatment strategies among serodiscordant couples, which found that early initiation of ART results in a 96% reduction in sexual transmission [14].

Conclusive evidence for HIV treatment as prevention (TasP) represents a major victory in the fight against HIV/AIDS. However, broad-scale implementation of this strategy is expected to be expensive and challenging [7], [15], [16]. A recent study that was conducted in rural China found no benefit of ART in reducing transmission in discordant couples, further highlighting the challenges to effective TasP implementation in the real world [17], [18].

In 2002, China launched its National Free Antiretroviral Treatment Program (NFATP), and the program has expanded rapidly nationwide since 2003. By the end of 2009, treatment coverage for eligible patients had increased to 63% and mortality had decreased to less than 20 deaths per 100 person-years (PY) [19], [20]. Coverage for eligible patients has since expanded even further to 74% by the end of 2011 [1]. Although there have been substantial improvements in the provision of treatment services to HIV-positive patients in China, challenges remain, which include the rapid increase in the number of HIV-positive individuals that progress from HIV infection to clinical AIDS stage, and the considerable numbers of HIV-infected individuals who are eligible for treatment but have not been provided with ART, particularly among HIV-discordant couples [1]. In this study, we aimed to test the hypothesis that China's current HIV treatment program prevents new infections among discordant, heterosexual couples.

## Methods

### Study site and participants

**Figure 1:**
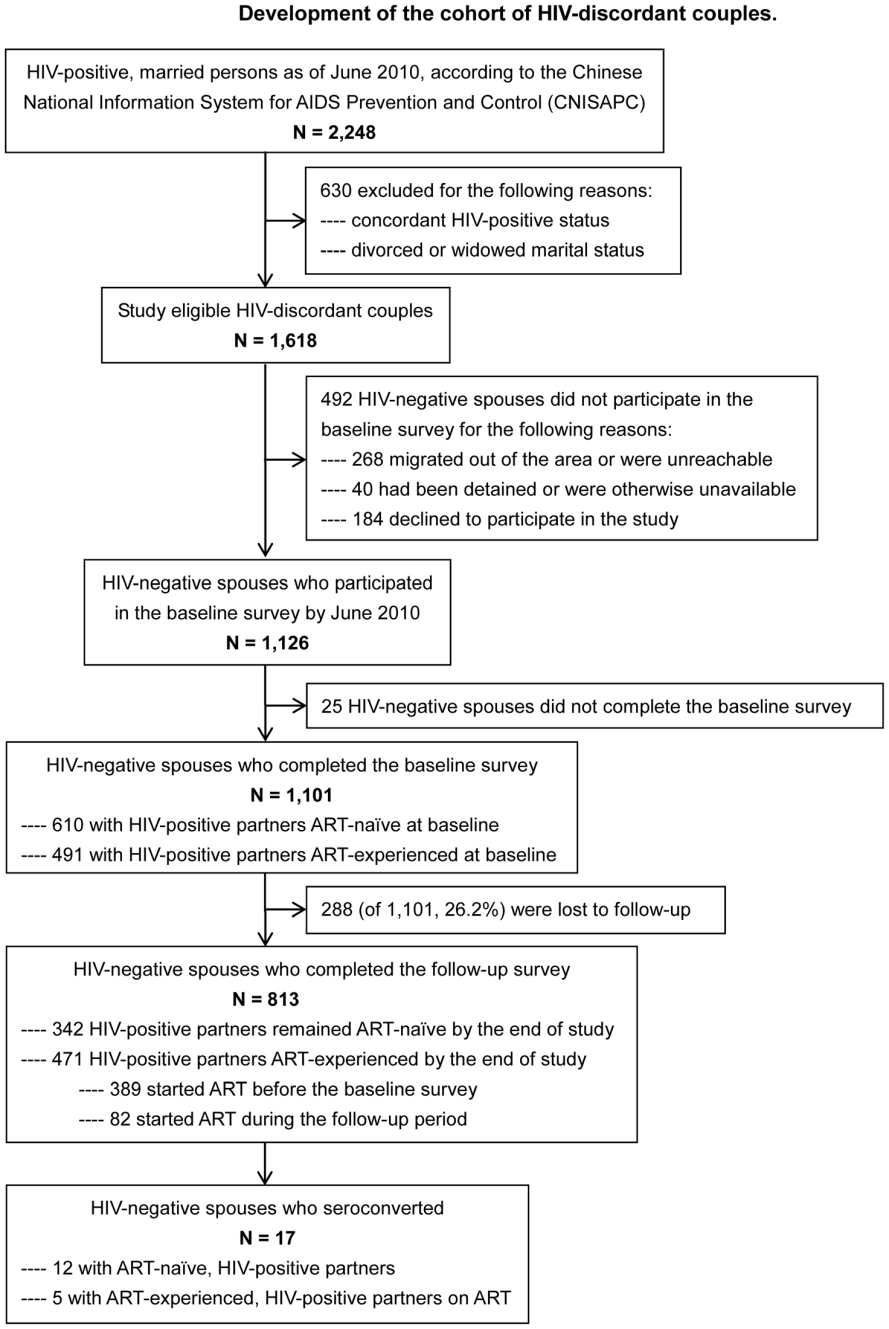
Flow chart depicting the development of the cohort of HIV-discordant couples who participated in this study of HIV discordant couples in rural Yunnan, China, 2009–2011.

A prospective, longitudinal cohort study that assessed the HIV incidence and its determinants among HIV-discordant couples was conducted from June 2009 to March 2011 in Dehong, Yunnan. Dehong is an autonomous prefecture in Western Yunnan bordering Myanmar that is one of the regions most affected by HIV in China [21], [22]. All previously identified HIV-discordant couples in Dehong were invited to participate in the study. Each participant was asked to return for one follow-up visit 12 months after the baseline survey, before March 2011. HIV-positive partners were eligible for the study if they were confirmed to a) have an AIDS diagnosis or HIV-positive status, b) be 18 years or older, c) reside in the selected town, and d) have a sero-negative spouse residing in the selected town. HIV-negative partners were eligible for the study if they were confirmed to a) have an HIV-negative status, b) be 18 years or older, c) reside in the selected town, and d) have a sero-positive spouse residing in the selected town. It should be noted that all HIV-negative partners have been informed of the positive status of the index partner within one month after confirmation of HIV-positive status, as a requirement of Yunnan Provincial HIV/AIDS Prevention and Treatment Regulation that was issued in 2006 [23]. A flow chart describing the development of our cohort of HIV-discordant couples can be found in **Figure 1**.

### Antiretroviral treatment

Routine ART was prescribed for HIV-positive partners in accordance with China's national guidelines, i.e., initiation of ART was primarily based on a patient's CD4 level or clinical stages, and none of the HIV-positive partners were treated specifically because of their serodiscordance status. Prior to 2008, the eligible criteria for initiating ART include all patients with CD4≤200 cells/µl, or patient reaching WHO clinical stage 3 or 4. The criteria were then modified to include those with CD4≤350 cells/µl in 2008 [20]. Therefore, some HIV-positive partners included in this study began ART earlier than others. Furthermore, some HIV-positive partners who were not on ART at baseline started ART during the follow-up period. Only those who started ART before the midpoint of the follow-up period were classified as ART-experienced group. Those who started ART after the midpoint of the follow-up period were still classified as ART-Naïve group, given the fact that it usually takes 3 to 6 months to achieve viral suppression among patients who take ART.

### Data and sample collection

At baseline and at follow-up visit, each participant was interviewed face-to-face by a trained and experienced public health professional in a private place using a structured questionnaire. Both HIV-positive and HIV-negative spouses were asked about their demographic characteristics and their sexual and drug use behaviors. Frequency of condom use with their HIV-positive partner in the past 12 months was indicated as “never”, “sometimes”, “nearly always”, or “every time” and recoded as inconsistent (never, sometimes, or nearly always) vs. consistent (every time). At each visit, 10 ml venous blood samples were collected from each participant. Plasma was extracted and stored at −70°C for later HIV-1, syphilis, herpes simplex virus 2 (HSV-2), CD4, and viral load testing as well as HIV genotype and phylogenetic analysis.

### HIV-1, syphilis, and HSV-2, CD4, and viral load testing

HIV testing was performed using a commercially-available ELISA (Kehua Biotech, China). All samples that screened positive for HIV were confirmed by Western blot (Genelabs Diagnostics, Singapore). Testing for syphilis was performed using a rapid plasma reagent (RPR) test (Kehua Biotech, China) with confirmation by *Treponema pallidum* particle agglutination assay (TPHA, Lizhu Biological Medicine, China). Subjects with plasma positive for syphilis in both RPR and TPHA tests were determined to be currently infected with syphilis. HSV-2 IgG antibody testing was performed using the Captia anti-HSV-2 IgG ELISA (Trinity Biotech, Ireland). CD4^+^ T cell counts were measured by FACSCount (Becton Dickinson, USA). HIV viral load was measured with the NucliSENS EasyQ HIV-1 assay (Biomérieux, France), which had a lower detection limit (LDL) of 50 copies/ml. All tests were performed according to manufacturers' instructions.

### HIV genotype and phylogenetic analysis

RNA extraction, RT-PCR amplification, and nucleotide sequencing were performed in physically separated laboratories. Viral RNA was extracted using the Viral RNA Extraction Mini Kit (Roche, USA). Extracted RNA was reverse-transcribed into cDNA, which was used as the template for PCR amplification of the HIV-1 *pol* gene by nested PCR (Takara Biotechnology, China). Primers and PCR conditions have been previously described [24]–[26]. PCR products were sequenced using the ABI PRISM 3730XL DNA Analyzer (Applied Biosystems, USA).

Nucleotide sequences of amplified *pol* regions from all HIV-positive participants together with other non-clustered control sequences were aligned using the ClustalX program in MEGA software version 5.0. Phylogenetic and molecular evolutionary analyses were conducted using MEGA software version 5.0. Evolutionary distances were calculated using Kimura two-parameter modeling, excluding positions with alignment gaps in any sequence. Phylogenetic dendrograms were constructed using the neighbor-joining method with Kimura two-parameter modeling. The reliability of each node was evaluated by bootstrapping with 2,000 replicates.

### Statistical analysis

Chi-square test and Fisher's exact test were used to compare categorical variables. HIV incidence was calculated by using the number of persons who seroconverted as the numerator and the amount of HIV-seronegative time in follow-up as the denominator. Those who did not seroconvert contributed follow-up time until the study end date. Those who were found to have seroconverted during follow-up were assumed to have seroconverted at the midpoint between baseline and follow-up visit, thus they contributed follow-up time until this midpoint date. Poisson 95% confidence intervals (CIs) were calculated for overall incidence and for subgroup comparisons.

Univariate and multivariate Cox proportional hazards regression models were used to examine factors associated with seroconversion. In multivariate analysis, we included demographic variables and those variables with p-values less than 0.1 in univariate analyses and with forced entry of HSV-2 status and frequency of sex in past 12 months. Plasma viral load and condom use variables were not adjusted in the multivariate model because they were most likely to be consequences of ART. A sensitivity analysis was performed by additionally adjusting multivariate regression models for the variable “plasma viral load”. The variable “Consistent condom use in past 12 months” was not included in the sensitivity analysis because no seroconverter was found among couples who reported “consistent condom use in past 12 months”. Because reported rates of condom use were so high, stratification analysis on condom use in the past 12 months at follow-up was conducted. All statistical analyses were performed using SAS software (Version 9.11, SAS Institute, USA).

### Ethics Statement

This study protocol, including design, subjects recruiting, consent procedure, etc., was reviewed and approved by the Institutional Review Board of the Chinese National Center for AIDS/STD Control and Prevention, Chinese Center for Disease Control and Prevention, written informed consents were obtained from all participants, with one copy given to the subject and one copy for study document. Each participant was given compensation of CNY60 (∼USD9), for their time at each study visit.

## Results

### Characteristics of the cohort at baseline

A total of 1,618 HIV-discordant couples were eligible for the study, 492 did not participate in the baseline survey and 25 did not complete the baseline survey, and a total of 1,101 HIV-negative spouses completed the baseline survey and were enrolled in the study. Of the 1,101 HIV-negative participants, 288 (26.2%) did not return for follow-up visits. Thus, 813 (73.8%) were successfully followed (**Figure 1**). As shown in **Table 1**, the two groups (“retained in cohort” *vs.* “lost to follow-up”) did not differ significantly with respect to most variables. However, of those who were lost to follow-up, a greater proportion of HIV-negative spouses reported less frequent sex in the past 12 months (p = 0.0002) and a greater proportion of HIV-positive partners were not receiving ART (p = 0.0003).

**Table 1:** Baseline characteristics of those retained in the cohort and lost to follow-up among study of HIV discordant couples in rural Yunnan, China, 2009–2011.

Characteristics	Retained in Cohort N (%)	Lost to Follow-up N (%)	P-value
***HIV-negative partners at baseline***			
Age (years)			
<30	233 (28.6)	80 (27.8)	0.9380
30–39	338 (41.6)	123 (42.7)	
≥40	242 (29.8)	85 (29.5)	
Gender			
Female	646 (79.5)	220 (76.4)	0.2746
Male	167 (20.5)	68 (23.6)	
Education			
Illiterate or primary school	560 (68.9)	203 (70.5)	0.6117
Junior/middle or high school	253 (31.1)	85 (29.5)	
History of any drug use			
No	787 (96.8)	279 (96.9)	0.9516
Yes	26 (3.2)	9 (3.2)	
History of injecting drug use			
No	808 (99.4)	285 (99.0)	0.4638
Yes	5 (0.6)	3 (1.0)	
Injecting drug use in past 12 months			
No	812 (99.9)	288 (100.0)	0.5515
Yes	1 (0.1)	0 (0.0)	
Frequency of sex in past 12 months (times/month)			
No sex	73 (9.1)	51 (18.2)	0.0002
1–3	314 (39.1)	99 (35.4)	
4–30	417 (51.9)	130 (46.4)	
Consistent condom use in past 12 months			
No sex	73 (9.1)	51 (18.2)	<0.0001
No	182 (22.2)	70 (25.0)	
Yes	549 (68.3)	159 (56.8)	
Syphilis serostatus			
Negative	752 (98.8)	253 (98.1)	0.3677
Positive	9 (1.2)	5 (1.9)	
HSV-2 serostatus			
Negative	527 (70.0)	185 (72.8)	0.3885
Positive	226 (30.0)	69 (27.2)	
***HIV-positive partners (Index Cases) at baseline***			
History of any drug use			
No	400 (51.9)	125 (49.8)	0.5569
Yes	371 (48.1)	126 (50.2)	
History of injecting drug use			
No	469 (60.8)	149 (59.4)	0.6796
Yes	302 (39.2)	102 (40.6)	
Injecting drug use in past 12 months			
No	741 (96.1)	234 (93.2)	0.0583
Yes	30 (3.9)	17 (6.8)	
Syphilis serostatus			
Negative	706 (96.8)	233 (98.3)	0.2339
Positive	23 (3.2)	4 (1.7)	
HSV-2 serostatus			
Negative	458 (63.6)	145 (62.2)	0.7042
Positive	262 (36.4)	88 (37.8)	
Antiretroviral treatment			
No	424 (52.2)	186 (64.6)	0.0003
Yes	389 (47.8)	102 (35.4)	
CD4^+^ T cell count (cells/µl)			
<200	108 (15.3)	38 (16.5)	0.9618
200–349	187 (26.4)	58 (25.2)	
350–499	170 (24.0)	56 (24.4)	
≥500	243 (34.3)	78 (33.9)	
Median (IQR)	399 (244–568)	396 (262–564)	
Plasma HIV viral load (copies/ml)			
<400	379 (51.9)	102 (43.4)	0.0719
400–9,999	220 (30.1)	80 (34.0)	
10,000–49,999	30 (4.1)	8 (3.4)	
≥50,000	101 (13.8)	45 (19.2)	
Overall	813 (100.0)	288 (100.0)	-

IQR: Inter-quartile range.

#### Characteristics of HIV-negative partners

Among those retained in the cohort, the majority of individuals were female (79.5%), aged 39 years or younger (70.2%), illiterate or educated at primary school only (68.9%), and had never used drugs (96.8%). At baseline, 90.9% of participants reported having had sex with their HIV-positive partner and 22.2% reported inconsistent condom use in the past 12 months. At baseline, 1.2% of HIV-negative partners were syphilis-positive, and 30% were HSV-2-positive (**Table 1**). Ten participants (1.2%) reported having had commercial sex in their lifetime and only two reported having had commercial sex in the past 12 months. Eleven participants (1.4%) reported having had non-commercial, extramarital sex in their lifetime and only one in the past 12 months.

#### Characteristics of HIV-positive partners (Index Cases)

History of drug use was reported by 48.1% of HIV-positive subjects and history of injecting drugs by 39.2%. A total of 30 HIV-positive subjects (3.9%) reported injecting drug use within the past 12 months. The prevalence of syphilis was 3.2% and the prevalence of HSV-2 was 36.4%. Among HIV-positive partners, 47.8% were already receiving ART at baseline. Median CD4 was 399 cells/µl (inter-quartile range [IQR]: 244–568 cells/µl). In terms of viral load, 51.9% had <400 copies/ml, 30.1% had 400–9,999 copies/ml, and 17.9% >10,000 copies/ml (See **Table 1**).

### Characteristics of the cohort at the follow-up

#### Characteristics of HIV-negative partners

As summarized in **Table 2**, 85.4% of participants reported having had sex with their HIV-positive spouse and 16.6% reported inconsistent condom use in the past 12 months (**Table 2**). Seventeen participants (2.1%) reported having bought commercial sex in their lifetime. Among them, only one reported having had commercial sex in the past 12 months. Seventeen participants (2.1%) also reported having had non-commercial, extramarital sex in their lifetime, and four reported having had non-commercial extramarital sex in the past 12 months. Ten participants (1.2%) reported having injected drugs in their lifetime, but none reported injecting drugs in the past 12 months.

**Table 2:** Comparison of characteristics of HIV-negative and HIV-positive partners at baseline and follow up relative to whether the HIV-positive partner (index case) was on ART or naïve to ART among study of HIV discordant couples in rural Yunnan, China, 2009–2011.

Characteristics	At Baseline*					At Follow-Up*				
	Overall N (%)	Index Case On ART N (%)	Index Case ART-NaïveN (%)	χ^2^-value	P-value	Overall N (%)	Index Case On ART N (%)	Index Case ART-NaïveN (%)	χ^2^-value	P-value
***HIV-negative partners***										
Frequency of sex in past 12 months (times/month)										
No sex	73 (9.1)	39 (10.1)	34 (8.1)	1.037	0.5953	119 (14.6)	51 (10.8)	68 (19.9)	13.117	0.0014
1–3	314 (39.3)	147 (38.1)	167 (40.0)			306 (37.6)	183 (38.9)	123 (36.0)		
4–30	417 (51.6)	200 (51.8)	217 (51.9)			388 (47.7)	237 (50.3)	151 (44.1)		
Consistent condom use in past 12 months										
No sex	73 (9.1)	39 (10.1)	34 (8.1)	35.616	<0.0001	119 (14.6)	51 (10.8)	68 (19.9)	20.085	<0.0001
No	182 (22.2)	52 (13.5)	130 (31.1)			135 (16.6)	68 (14.4)	67 (19.6)		
Yes	549 (68.3)	295 (76.4)	254 (60.8)			559 (68.8)	352 (74.7)	207 (60.5)		
***HIV-positive partners (Index Cases)***										
CD4^+^ T cell count (cells/µl)										
<200	108 (15.3)	54 (15.6)	54 (15.0)	17.935	0.0005	70 (11.6)	43 (11.2)	27 (12.4)	1.397	0.7062
200–349	187 (26.4)	111 (32.0)	76 (21.0)			126 (20.9)	86 (22.3)	40 (18.3)		
350–499	170 (24.0)	87 (25.1)	83 (23.0)			119 (19.7)	75 (19.5)	44 (20.2)		
≥500	243 (34.3)	95 (27.3)	148 (41.0)			288 (47.8)	181 (47.0)	107 (49.1)		
Median (IQR)	399 (244–568)	361 (252–515)	431 (276–631)			473 (303–674)	464 (303–675)	489 (316–658)		
Plasma HIV viral load (copies/ml)										
<400	379 (51.9)	307 (85.0)	72 (19.5)	313.979	<0.0001	375 (62.8)	318 (82.6)	57 (26.9)	186.028	<0.0001
400–9,999	220 (30.1)	35 (9.7)	185 (50.1)			132 (22.1)	42 (10.9)	90 (42.4)		
10,000–49,999	30 (4.1)	4 (1.1)	26 (7.1)			21 (3.5)	2 (0.5)	19 (9.0)		
≥50,000	101 (13.8)	15 (4.2)	86 (23.3)			69 (11.6)	23 (6.0)	46 (21.7)		
Overall	813 (100)	389 (47.8)	424 (52.2)	-	-	813 (100)	471 (57.9)	342 (42.1)	-	-

IQR: Inter-quartile range.

* A total of 82 HIV-positive partners (index cases) began ART during the follow-up period. At baseline, these individuals were included in the “Naïve to ART” category and at follow-up, they were included in the “On ART” category.

#### Characteristics of HIV-positive partners (Index Cases)

At the follow-up visit, median CD4 of HIV-positive partners overall was 473 cells/µl (IQR: 303–674 cells/µl). In terms of viral load, 62.8% had <400 copies/ml, while 22.1% had 400–9,999 copies/ml and 15.1% had >10,000 copies/ml. A total of 471 (57.9%) HIV-positive partners were receiving ART at follow-up, 82 initiated ART during the follow-up period (See **Table 2**).

### Comparison of discordant couples receiving and not receiving ART

As summarized in **Table 2**, frequency of sex in the past 12 months (p = 0.0014, follow-up) and consistent condom use in the past 12 months (p<0.0001, baseline and follow-up), were greater among couples given that the HIV-positive partner was ART-experienced. Those partners who were on ART already at baseline were more likely to have CD4>350 cells/µl than those naïve to ART (p = 0.0005, baseline). HIV-positive partners on ART were more likely to have viral load <400 copies/ml (p<0.0001, baseline and follow-up).

### HIV seroconversion among HIV-negative participants

The average follow-up time for the 813 HIV-negative participants retained in the cohort was 1.4 PY. As summarized in **Table 3**, a total of 17 seroconversions were documented during a total of 1,127 PY of follow-up, resulting in an overall incidence of 1.5 per 100 PY. None of these seroconverters were ever injecting drug users, nor had they reported having had extramarital sex in the past 12 months. HIV genotyping and phylogenetic analysis for the 17 seroconverters and their HIV-infected spouses indicated that the partners of each of the 17 HIV-positive couples not only had the same HIV subtype, but that their viral sequences clustered together in phylogenetic tree analysis. HIV incidence was significantly higher among participants who reported inconsistent condom use (9.7 per 100 PY), and who had HIV-positive partners with viral load >400 copies/ml (2.3 per 100 PY for 400–9,999 copies/ml, 5.0 per 100 PY for 10,000–49,000 copies/ml, 5.1 per 100 PY for >50,000 copies/ml) or had an HIV-positive partner who was not receiving ART (2.4 per 100 PY).

**Table 3:** Univariate and multivariate analyses of risk factors for HIV seroconversion among HIV-negative participants during follow-up HIV discordant couples in rural Yunnan, China, 2009–2011.

Variables	SeroconvertersN (%)	PY	HIV Incidence (per 100 PY)	Unadjusted HR (CI)	P-value	Adjusted HR (CI)	P-value
***Variables of HIV-negative partners at baseline***							
Age (years)							
<30	8 (47.0)	310	2.6	1.00		1.00	
30–39	7 (41.2)	470	1.5	0.57 (0.21–1.58)	0.2806	0.53 (0.19–1.50)	0.2316
≥40	2 (11.8)	348	0.6	0.22 (0.05–1.02)	0.0536	0.30 (0.06–1.52)	0.1479
Gender							
Female	12 (70.6)	901	1.3	1.00		1.00	
Male	5 (29.4)	226	2.2	1.64 (0.58–4.65)	0.3529	1.82 (0.59–5.66)	0.2985
Education							
Illiterate or primary school	12 (70.6)	781	1.5	1.00		1.00	
Junior/middle or high school	5 (29.4)	346	1.4	0.94 (0.33–2.66)	0.9016	0.78 (0.26–2.32)	0.6551
Syphilis serostatus							
Negative	17 (100.0)	1071	1.6	1.00			
Positive	0 (0.0)	13	0.0	0.00 (0.00-0.00)	0.5144		
HSV-2 serostatus							
Negative	12 (70.6)	757	1.6	1.00		1.00	
Positive	5 (29.4)	317	1.6	1.00 (0.35–2.84)	1.0000	1.14 (0.34–3.86)	1.0000
***Variables of HIV-positive partners (Index Cases) at baseline***							
Syphilis serostatus							
Negative	17 (100.0)	999	1.7	1.00			
Positive	0 (0.0)	33	0.0	0.00 (0.00-0.00)	0.2900		
HSV-2 serostatus							
Negative	11 (64.7)	648	1.7	1.00		1.00	
Positive	6 (35.3)	370	1.6	0.95 (0.35–2.58)	0.9254	0.74 (0.22–2.46)	0.6186
CD4^+^ T cell count (cells/µl)							
<200	2 (11.8)	152	1.3	1.00			
200–349	3 (17.7)	266	1.1	0.86 (0.14–5.17)	0.8732		
350–499	6 (35.3)	242	2.5	1.85 (0.37–19.16)	0.4512		
≥500	6 (35.3)	345	1.7	1.32 (0.27–6.55)	0.7327		
Plasma HIV viral load (copies/ml)							
<400	1 (5.9)	552	0.2	1.00			
400–9,999	7 (41.2)	305	2.3	12.89 (1.59–104.78)	0.0168		
10,000–49,999	2 (11.8)	40	5.0	27.29 (2.47–301.00)	0.0069		
≥50,000	7 (41.2)	138	5.1	27.49 (3.38–223.39)	0.0019		
***Variables of HIV-negative partners at follow-up***							
Frequency of sex in past 12 months (times/month)†							
1–3	5 (29.4)	435	1.1	1.00		1.00	
4–30	12 (70.6)	524	2.3	2.01 (0.71–5.70)	0.1896	1.72 (0.59–5.01)	0.3174
Consistent condom use in past 12 months†							
No	17 (100.0)	175	9.7	1.00			
Yes	0 (0.0)	784	0.0	0.00 (0.00-0.00)	<0.0001		
***Variables of HIV-positive partners (Index Cases) at follow-up***							
ART for HIV-positive partners*							
No	12 (70.6)	505	2.4	1.00		1.00	
Yes	5 (29.4)	622	0.8	0.34 (0.12–0.97)	0.0436	0.30 (0.10–0.86)	0.0250
Overall	17 (100.0)	1,127	1.5	-	-	-	-

PY: Person years; HR: Hazard ratio; CI: Confidence interval (95%).

* Only those already on ART at baseline or started ART before the midpoint were considered to be receiving ART for the purposes of univariate and multivariate analyses.

† “No sex” category was excluded because there were no seroconverters among participants who reported having had no sex with spouses in past 12 months at follow-up.

### ART, condom use, and HIV seroconversion

Study participants whose HIV-positive partner was receiving ART had a significantly lower rate of HIV seroconversion compared to those with partners who were not receiving ART in both univariate analysis (HR = 0.34, CI: 0.12–0.97; p = 0.0436) and multivariate analysis (HR = 0.30, CI: 0.10–0.86; p = 0.0250) after adjusting for potential confounding variables using Cox proportional hazard model (**Table 3**). The sensitivity analysis with additional adjustment for plasma viral load showed that the association between ART and HIV seroconversion was not statistically significant (p = 0.6387) (Data not shown).

Stratification analysis of the variable consistent condom use during the past 12 months at follow-up showed that among the 135 participants (175 PY) who reported having had sex without consistent condom use during the past 12 months at follow-up, HIV incidence was 13.0 per 100 PY for those whose HIV-positive partners were not receiving ART, two times higher than those whose partners were receiving ART (6.0 per 100 PY, p = 0.1371) (Table 4). A total of five of 17 seroconversions observed in this study (29.0%), occurred in couples in which the HIV-positive partner was on ART prior to baseline. The characteristics of these couples at both baseline and follow-up are summarized in Table 5. All reported inconsistent condom use and four HIV-positive partners had viral load >10,000 copies/ml at baseline.

**Table 4:** Stratification analysis of consistent condom use in the past 12 months at follow-up survey among study of HIV discordant couples in rural Yunnan, China, 2009–2011.

	Condom use during the past 12 months at follow-up									
	No Sex (n_1_ = 119)			Inconsistent Condom Use (n_2_ = 135)				Consistent Condom Use (n_3_ = 559)		
	Sero- converters	Observed Time (PY)	HIV Incidence (per 100 PY)	Sero- converters	Observed Time (PY)	HIV Incidence (per 100 PY)	P-value	Sero- converters	Observed Time (PY)	HIV Incidence (per 100 PY)
ART for HIV-positive partners*										
No	0	97	0.0	12	92	13.0	0.1371	0	316	0.0
Yes	0	71	0.0	5	83	6.0		0	468	0.0

PY: Person-years.

* Only those already on ART at baseline or started ART before the midpoint were considered to be receiving ART for the purposes of stratification analyses.

**Table 5:** Characteristics of the five individuals who seroconverted despite their HIV-positive spouse receiving ART among study of HIV discordant couples in rural Yunnan, China, 2009–2011.

Characteristics*	Subject 1		Subject 2		Subject 3		Subject 4		Subject 5	
	Baseline	Follow-up	Baseline	Follow-up	Baseline	Follow-up	Baseline	Follow-up	Baseline	Follow-up
***Variables of seroconverted spouses***										
Age (years)	38	39	34	34	33	34	37	38	36	37
Gender	Female		Male		Female		Female		Female	
Education	Illiterate or primary		Junior/middle or high		Illiterate or primary		Illiterate or primary		Illiterate or primary	
Frequency of sex† (times per month)	3	3	15	4	7	6	5	7	2	10
Consistent condom use‡	Yes	No	Yes	No	No	No	Yes	No	Yes	No
Syphilis serostatus	Negative	Negative	Negative	Negative	Negative	Negative	Negative	Negative	Negative	Negative
HSV-2 serostatus	Negative	Negative	Negative	Negative	Positive	Positive	Negative	Negative	Positive	Positive
***Variables of HIV-positive spouses (Index Cases)***										
Syphilis serostatus	Negative	Negative	Negative	Negative	Negative	Negative	Negative	Negative	Negative	Negative
HSV-2 serostatus	Negative	Negative	Negative	Negative	Positive	Positive	Negative	Negative	Negative	Negative
Antiretroviral treatment§	Yes	Yes	Yes	Yes	Yes	Yes	Yes	Yes	Yes	Yes
CD4^+^ T cell count (cells/µl)	382	391	470	282	259	329	380	311	111	82
Plasma HIV viral load (copies/ml)	11,000	760,000	12,000	14,000	43,000	<LDL	67,000	<LDL	<LDL	500,000

LDL: lower detection limit, which for this study was 50 copies/ml.

* All 5 seroconverters answered no to commercial sex, non-commercial sex, and injection drug use questions for both lifetime and most recent 12-month time periods, thus this data was not included in the table.

† Frequency of sex variable related to sex with spouses within the past 12 months.

‡ Consistent condom use variable related to sex with spouses within the past 12 months.

§ All HIV-positive spouses of the 5 seroconverters were on ART already at baseline.

## Discussion

We have shown that uninfected partners of HIV-positive patients receiving ART have approximately one third of the risk of being infected with HIV compared to those of HIV-positive patients not receiving ART. This finding is consistent with studies from other regions that have indicated effective ART can greatly reduce the likelihood of HIV sexual transmission from infected individuals to their sexual partners [5], [8]–[14]. We find that the HIV incidence among discordant couples who are not receiving ART (2.4 per 100 PY) to be similar to that of injecting drug users (2.3 per 100 PY) [27] and men who have sex with men (2.6 per 100 PY) [28] in China, which suggests that HIV-negative spouses of HIV-positive patients are a similarly high-risk population. With the application of regular HIV treatment in this population, we realized a 66% reduction in incidence to 0.8 per 100 PY. The overall incidence of HIV infection among seronegative partners was 1.5 per 100 PY, similar to what was found by Wang *et al.* (1.7 per 100 PY) [17], and in the HPTN 052 study (1.2 per 100 PY) [14], but lower than the incidence rates among discordant couples recently reported in Africa (4.3 per 100 PY and 9.2 per 100 PY) [5], [11]. The relatively low overall HIV incidence in our sample may be attributed to higher condom use, lower prevalence of STIs, lower prevalence of high-risk sexual behavior, and/or greater availability of ART.

Our findings provide important new evidence for regular treatment as a prevention strategy and are in direct opposition to the study that previously reported no benefits of ART on reducing HIV seroconversion in China [17]. There are at least two possible explanations for this disparity. First, approximately 80% of the HIV-positive partners in the study by Wang *et al.* were receiving ART, which may have been a sufficiently high percentage to reduce the power of their statistical analysis. Second, the previous study did not present data on viral load of individuals in their sample population. This may result in miss-classification of patients as receiving effective ART, when in reality ART adherence may be low and/or ineffectively suppressing viral load [29]. Despite its limitations, Wang's study did point out major obstacles to the successful implementation of Treatment as Prevention approach in China, including the difficulties of ensuring consistent, long-term viral suppression, the provision of on-going monitoring of viral load, and the thorough examination of virological failure cases [17], [18]. Another challenge we encountered in our study was the high proportion of lost to follow up in the non-ART group. We believe that the issue of the missing group of HIV discordant couples can be partially addressed by initiating ART and/or treatment as prevention early, thereby reducing the risks of HIV transmission.

We found that of the five cases of HIV transmission in couples in which the infected partner was receiving ART, four of the infected partners of these seroconverters had viral load >10,000 copies/ml, suggesting that virological failure may have resulted in these transmission events. Seroconversions of HIV-negative partners despite the HIV-positive partner receiving ART have thus far been observed only in rare instances and have been suggested to perhaps be the result of incomplete suppression of viral load in semen or cervicovaginal secretions [7], [10], [14]. We also noted even among those on ART, there were no obvious changes in CD4 cell counts and plasma HIV viral load from baseline to follow-up visit, suggesting that some of them might have undergone immunologic or virological failure, or had low adherence to ART. These findings highlight the need to regularly monitor the viral load, drug resistance, and adherence counseling status among HIV-positive patients who are receiving ART.

While it is well known that the risk of HIV transmission per coital act depends on a number of behavioral and biological factors, prevention efforts targeted specifically at each of these factors have generated mixed results over the past thirty years [30]. For example, sexual abstinence education has been proved to be ineffective among discordant couples, and many of them would choose to continue to have a normal sexual and reproductive life [31]. Also, while condom use are is proven to be effective in reducing transmission risk [32], the rate of condom use remains low among couples in stable relationships [5], [33], [34]. As our results indicated, all of the five seroconverters who had an infected partner on ART reported no consistent condom use at follow-up visit, which highlights the importance of emphasizing safe sex education for these HIV-discordant couples, even if the HIV-Positive partner is already on treatment. On the other hand, in the present study, a very high proportion of participants continued to maintain a normal sex life and reported consistency in using condoms. This effect underscores the differences in efficacy between early ART as TasP in controlled clinical trials [14], and regular treatment in practice.

Our study has several limitations. Firstly, we included people engaged in stable, heterosexual, HIV-discordant relationships in rural China, which may be neither representative nor generalizable. Secondly, viral load was only collected and measured at baseline and follow-up, not allowing for observations of changes over time. Thirdly, while none of the seroconverters reported having extramarital sex or injecting drug use, there was no effective measure in place to assess the validity of their claims, and the likelihood of misreporting was not estimated. However, it should be noted that genotype and phylogenetic linkage analysis confirms the partners as the source of infection in our study. Fourthly, questions included in the survey on risk behaviors were limited to HIV-related risk behaviors a participant may have engaged in over the past 12 months, some of the participants did not return for a follow up visit for up to 21 months after baseline. This means there could be up to 9 months of follow-up time which would be potentially left unaccounted for, resulting in information bias. Given this was a relatively short term follow-up study, we assumed that the practice of sexual behaviors among couples, especially the use of condoms, followed a regular pattern during the study period; and will have minimum impact on the study results. Also, couples who reported having no sex in the past 12 months could be due to that crackdowns on illicit drug use detained some HIV positive partners for large periods of the observation period. Finally, because of the relatively short follow-up period, we cannot determine the sustainability of ART-induced reductions in HIV transmission over the longer term.

Our study represents a test of the real-life benefit of regular HIV treatment for preventing new HIV infections in serodiscordant couples in rural areas. Despite the fact that we have highlighted some of the major challenges China is faced with in treating and preventing HIV infection, our finding of a 66% reduction in HIV transmission provides compelling evidence in support of implementing of HIV TasP. Application of the findings presented in the study will not only be critical for China as it further develops and expands its national ART program, but will also serve as an example of the effectiveness of this strategy for other developing nations.
